# An Effective Electric Dipole Model for Voltage-induced Gating Mechanism of Lysenin

**DOI:** 10.1038/s41598-019-47725-0

**Published:** 2019-08-07

**Authors:** Radwan Al Faouri, Eric Krueger, Vivek Govind Kumar, Daniel Fologea, David Straub, Hanan Alismail, Qusay Alfaori, Alicia Kight, Jess Ray, Ralph Henry, Mahmoud Moradi, Gregory Salamo

**Affiliations:** 1 Division of Sciences and Mathematics, University of the Ozarks, Clarksville, AR 72830 USA; 2 Department of Physics, Boise State University, Boise, ID 83725 USA; 3 Department of Chemistry and Biochemistry, University of Arkansas, Fayetteville, AR 72701 USA; 4 Department of Biochemistry and Molecular Biology, School of Medicine, University of Arkansas for Medical Sciences, Little Rock, AR 72205 USA; 5 King Abdullah International Medical Research Center, Applied Medical Sciences, King Saud University, Riyadh, Saudi Arabia; 6 Department of Biomedical Engineering, University of Arkansas, Fayetteville, AR 72701 USA; 7 Department of Biological Sciences, University of Arkansas, Fayetteville, AR 72701 USA; 8 Department of Physics, University of Arkansas, Fayetteville, AR 72701 USA

**Keywords:** Molecular modelling, Computational biophysics, Permeation and transport

## Abstract

Lysenin is a pore-forming toxin, which self-inserts open channels into sphingomyelin containing membranes and is known to be voltage regulated. The mechanistic details of its voltage gating mechanism, however, remains elusive despite much recent efforts. Here, we have employed a novel combination of experimental and computational techniques to examine a model for voltage gating, that is based on the existence of an “effective electric dipole” inspired by recent reported structures of lysenin. We support this mechanism by the observations that (i) the charge-reversal and neutralization substitutions in lysenin result in changing its electrical gating properties by modifying the strength of the dipole, and (ii) an increase in the viscosity of the solvent increases the drag force and slows down the gating. In addition, our molecular dynamics (MD) simulations of membrane-embedded lysenin provide a mechanistic picture for lysenin conformational changes, which reveals, for the first time, the existence of a lipid-dependent bulge region in the pore-forming module of lysenin, which may explain the gating mechanism of lysenin at a molecular level.

## Introduction

Channels represent a fundamental part of cellular membranes, which transport ions and molecules across the cell. Their three-dimensional architectures often allow for a selective passage of the transported species across the hydrophobic lipid barriers^1^. While the channel architecture and the chemical details of the channel pore governs the selectivity of the channel, the gating can be accomplished either by blocking the pathway or due to conformational changes that effectively open or close the channel. The mechanisms by which channels gate in response to cellular signals are of great interest due to their fundamental role in establishing and maintaining transmembrane potentials, cell signaling, cell-cell communication, and information processing^1–5^. Moreover, channel dysfunction is known to cause neurological and cardiac disorders^6,7^, and many biological toxins explicitly target protein channels^8,9^. Consequently, by understanding the mechanisms involved in channel gating, it becomes possible to adapt or modify channels to exhibit desired and controllable properties. This ability would enhance the potential to develop vehicles that deliver or release medication, remove toxic material, or simply respond to a specific stimulus. As a result, there has been intense interest in voltage gated ion channels studies which have already revealed structural elements whose function is to transition or switch the channel conduction state^10–15^. However, further studies are needed to uncover and understand the underlying mechanisms that govern ion channels, especially for a family of ion conducting pore-forming proteins found in a diverse set of eukaryotes and prokaryotes and the aerolysin β-pore forming toxins (aβ-PFTs)^16^, of which *lysenin* is a member.

Lysenin is a pore-forming protein from the coelomic fluid of the earthworm *E. fetida* which self-assembles and then inserts as ~3 nm diameter oligomeric channels in bilayer lipid membranes containing sphingomyelin. Its voltage regulation coupled with a high transport rate makes lysenin *useful as a model* for studying the underlying mechanisms that govern ion channels^17,18^. Recent Cryo-EM^19^ and crystal^20^ structural studies of lysenin pore have opened opportunities to increase understanding by uncovering the structure of the lysenin pore, which is made up of nine identical monomers inserted into the membrane in a β-barrel configuration, about 11 nm long and 12 nm wide, with an inner diameter that ranges from 1.6 to 2.5 nm. In addition, the N-terminal domain of lysenin has two domains, one of which forms the lumen of the pore and is called the pore-forming module (PFM) and the other is located at the mouth of the pore and is called the cap domain. The hydrophilic C terminus (receptor-binding) domain of the protein is hinged to the N-terminal cap domain and is arranged around one end of the pore, extending about 6 nm above the membrane. Lysenin self-insertion properties circumvent the inherent difficulties of purification, reconstitution, and limited stability of ion channels, while still exhibiting important properties such as voltage-induced and ligand-induced gating, pH and temperature sensitivity, and hysteresis in conductance^17,18,21,22^. Lysenin’s well-characterized behavior along with its stability in both water and membrane enhances the opportunity to explore the gating mechanism of this ion channel.

Previous work with ion channels has shown that a voltage sensitive domain on the protein is responsible for gating^2,23^. As a result, lysenin channels are expected to possess voltage sensitive domains that would interact with applied electric fields to trigger a conformational change in the channel that changes its ionic conductance. Key to our understanding has been the recent Cryo- EM structure and high-resolution crystal structure of the lysenin monomer bound to sphingomyelin to form a membrane pore.

With this knowledge and our observations of the lysenin gating behavior, we hypothesize that the mechanism of channel gating depends on (i) the charge distribution of critical amino acids that are forming an “effective electric dipole” and (ii) the flexibility of the N-terminal cap domains (collar) and C terminal domains of the nonamer. This research engineers the lysenin pores for specific transport applications to understand the function of structurally related pore forming proteins. The new discovery of the lipid-dependent bulge that we have noticed in the lumen of the channel may play a major role in both voltage and ligand-induced gating of lysenin as a model of ion channels.

## Results

**Figure 1:**
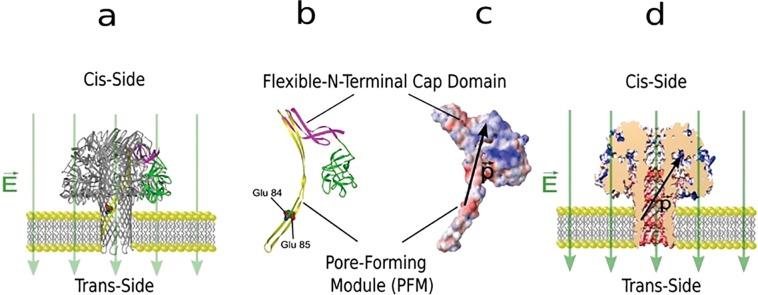
Lysenin structure and its electrostatic potential map; Lysenin is inserted in a BLM that is buffered by 130 mM KCl, 20 mM HEPES pH 7.0 (**a**) Cartoon representation of the lysenin pore in its open state. (**b**) Secondary structure of lysenin showing Glu84 and Glu85. (**c**) Electrostatic surface representation of lysenin shows the +5 kTe^−1^ positive potential regions (blue) at the flexible N-terminal cap domain, the −5 kTe^−1^ negative potential regions (red) at the PFM of the N-terminal domain, the hypothesized electric dipole (black). (**d**) Cross sectional view of lysenin pore showing the oriented effective electric dipole drawn from the middle of the negative region to the middle of the positive region.

Recent work on how lysenin forms a pore in a bilayer lipid membrane (BLM) containing sphingomyelin has revealed that lysenin subunits self-assemble on the surface of the sphingomyelin and then insert as a unit to form a pore^19,20^. Once inserted, however, published structure and images of the lysenin channel indicate that the lysenin pore is a nonamer^20^ (Fig. 1a). The crystal structure of monomer lysenin bound to sphingomyelin^19,20,24^ also predicts the orientation of each lysenin subunit in the BLM following channel assembly where the hydrophobic sphingosine-binding groove of the pore-forming module (PFM) of each subunit binds to the lipid environment to form the channel.

Our results support a model in which an effective internal electric dipole within lysenin N-terminus interacts with an applied electric field across the pore, inducing a torque on the dipole that aligns the dipole along the direction of the applied field, causing motion that closes the pore. In this model, the glutamates at positions Glu84 and Glu85 (Fig. 1a,b) contribute significantly to the total fixed negative charge in this region. An electrostatic surface representation of the lysenin monomer (Fig. 1c) shows the distribution of the fixed charges and uncovers additional physical features of the channel structure that have guided our mechanistic investigation of the channel gating mechanism. The N-terminus PFM contains fixed-negatively charged zones (red) close to the trans-side, where the N-terminal cap domain (collar) contains a fixed-positively charged zones (blue) exposed to the cis-side of the membrane (Fig. 1c). Consequently, negatively charged residues Glu84 & Glu85 play a role in the total strength of the “effective electric dipole” along the N-terminal domain. The Glu 84 residue faces the ion-conducting inner wall of the lysenin channel and Glu 85 is located on the other side (Fig. 1b–d); both residues are just above the membrane surface.

Our proposed voltage gating mechanism is supported by the following observations: (A) The recombinant lysenin protein retains its voltage gating functionality similar to the wild-type protein; (B) Engineering the recombinant lysenin protein successfully modifies the strength of the effective electric dipole, thereby changing the electrical gating properties of lysenin channels; (C) Increasing the viscosity of the solution increases the solvent drag force, slowing the gating; and (D) All-atom MD simulations elucidates the dependence of the conformational dynamics of lysenin on the strength of the proposed effective electric dipole at a molecular level. These investigations are discussed in detail in the following sections.

### Recombinant lysenin with affinity tags at each terminus retains normal voltage gating

**Figure 2:**
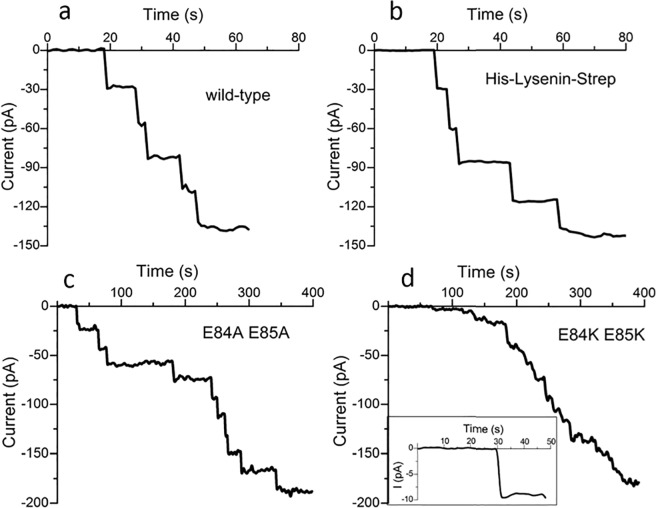
Channel insertion **c**omparison of wild-type lysenin with recombinant expressed lysenin and mutant lysenin. (**a**) Channels insertion of the wild-type. (**b**) Recombinant lysenin yield channels of similar conductance suggesting both channels are identical. (**c**) The lysenin mutant, E84A E85A produces discrete uniform channels into a planar BLM with an open current of ~20 pA/channel at −60 mV. (**d**) The fast insertion from the experimental setup hides the discrete channel insertions from the mutant E84K E85K, but single channel acquisition (**d** inset) reveals an open current of ~9 pA/channel at −60 mV. The full current-time graph that shows the insertion of a population of channels in the BLM is found in the Supplemental data (Supplementary Fig. S1).

Our idea was to modify the lysenin protein to explore the role of the effective electric dipole in gating. To do this we planned to make the recombinant expression of lysenin, demonstrate that it is a viable pore-forming protein^25–29^, and characterize its behavior under voltage gating. We have successfully produced lysenin by recombinant expression, and our purified protein contains a streptavidin tag (strep-tag II) on the C-terminus (GLY297) and a histidine tag on the N-terminus (MET1). Comparing the lysenin produced from recombinant expression with the extracted wild- type purchased from Sigma yielded identical channel insertions of ~30 pA/channel (Fig. 2a,b) when the membrane is voltage-clamped by −60 mV.

### Engineering lysenin by mutation to modify the strength of the effective electric dipole

The excellent results of the recombinant lysenin encouraged us to go further and modify the charge on the protein by amino acid substitution and therefore the effective electric dipole strength of the protein. Our opportunity to test our model is more than just exploring the motion of the flexible parts of the protein. For this investigation two modified lysenin proteins were produced. The two glutamates at positions 84 and 85 (Fig. 1b) are proposed to be part of the negative pole region of the lysenin effective electric dipole. They were replaced by two alanine residues to reduce the strength of the negative end of the dipole, by decreasing the negative charge in this area, while not altering the proposed movable part of the protein (N-terminal cap and C-terminal domains). Then, two lysines were substituted to further reduce the magnitude of the effective electric dipole. Each of the modifications yielded a protein which inserted as channels and could be explored for the effect on gating. Accordingly, any changes observed in the electrophysiological data would indicate the role that the substituted part played in voltage gating of the pore.

As expected, the E84A- E85A mutant, when exposed to planar lipid membranes in identical conditions to those of the wild-type, produced channels with a considerably lower conductance of ~20 pA/channel at −60 mV bias potential (Fig. 2c). To investigate this further, a double mutant of E84 E85 with lysine was generated. In identical electrophysiological conditions, the E84K E85K mutant produced channels in the membrane with ~50% less conductance than the E84A E85A mutant (Fig. 2d). The discrete insertions of the channels produce such a low burst of current that they are difficult to distinguish, but analysis of a single channel reveals an open current as low as ~9 pA/channel (inset, Fig. 2d).

### Increased solution viscosity plays a role in gating response

**Figure 3:**
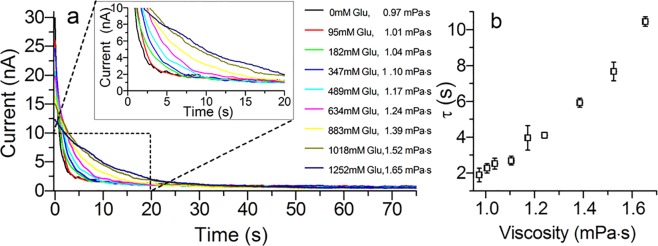
Increased electrolyte viscosity restricts channel closing. (**a**) Increasing concentrations of glucose gradually from 0 mM to 1.25 M increased the solution viscosity and slowed the channel closing for a constant +60 mV bias potential. All the channels exhibited complete closing after sufficient time. (**b**) Fitting the currents shown in (**a**) as an exponential decay yielded an increased time-constant for increased viscosity.

To explore the role of the flexible N-terminal cap (collar) and the C-terminus of the protein in gating, we restricted the motion of the N-terminal cap domain and the C-terminus by changing the viscosity of the electrolyte solution. Using wild-type lysenin channels inserted in planar BLMs at room temperature (~22 °C), the viscosity of the electrolyte solution was increased by the addition of small amounts of a 2 M glucose solution in 130 mM KCl, 20 mM HEPES pH 7.0 to both sides of the membrane to avoid differential osmotic effects. Glucose was added to the bathed solution over the range of 0–1.25 mM. After each addition of glucose, we allow the system to equilibrate, signaled by a constant ionic current. The time evolution of the current was recorded during the application of a trans-side constant potential of +60 mV. The increased glucose concentrations increased the time required to close the lysenin channels, shown in Fig. 3a. The resulting closing kinetics were fitted as a single exponential curve to yield the time constants for the time-dependent current as a function of solution viscosity (Fig. 3b). Although, the addition of glucose to the solution would also reduce the conductivity^30,31^ of the solution, our results show that the increased viscosity restricts the movement of N-terminal cap and the C- terminus domains increasing the time the channels remain in the open state. This behavior is also exhibited in the associated I-V curves by the shifted critical voltage for the increased viscosities (Supplementary Fig. S2).

### Molecular dynamics simulations

**Figure 4:**
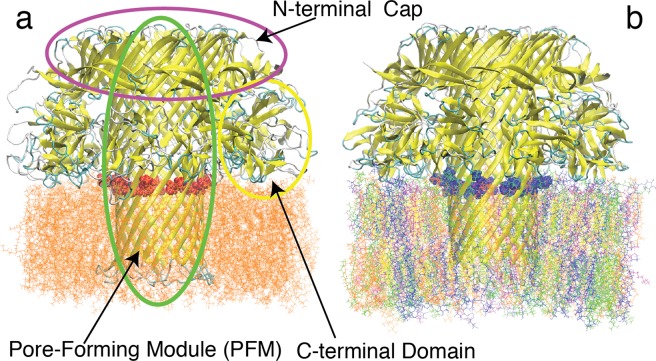
The cartoon representation of membrane-embedded (**a**) wild-type lysenin in a pure POPC lipid bilayer used in MD simulations. E84A and E85A residues are shown in the van der Waals surface representation (colored red). (**b**) E84K-E85K lysenin mutant in a mixed lipid bilayer consisting of POPC (orange), POPI (blue), POPE (magenta) CHOL (yellow), and SM (green). E84K and E85K residues are shown in the van der Waals surface representation (colored blue).

The MD simulations involved a nonameric lysenin pore, which was embedded in an explicit lipid bilayer. Two different lipid environments were modeled; one composed of pure POPC (1-palmitoyl-2-oleoyl-sn-glycero-3-phosphocholine) lipids (Fig. 4a), and the other composed of a mixture of lipids mimicking the composition used in our experiments (see Materials and Methods) including Asolectin from soybean, sphingomyelin (SM), and cholesterol (CHOL) (Fig. 4b). Asolectin mixture was represented by a combination of POPC, POPI (1-palmitoyl-2- oleoyl-sn-glycero-3-phosphoinositol), and POPE (1-palmitoyl-2-oleoyl-sn-glycero-3- phosphoethanolamine) lipids. This protein-membrane complex was solvated in a water box and equilibrated to determine the stability and fluctuations of different regions of the protein. Three different models of the lysenin were generated based on its recent cryo-EM structure^19^ including a wild-type (Fig. 4a), an E84A E85A mutant, and an E84K E85K mutant (Fig. 4b) lysenin. After energy minimization and initial relaxation, each system was simulated for 40 nanoseconds.

**Figure 5:**
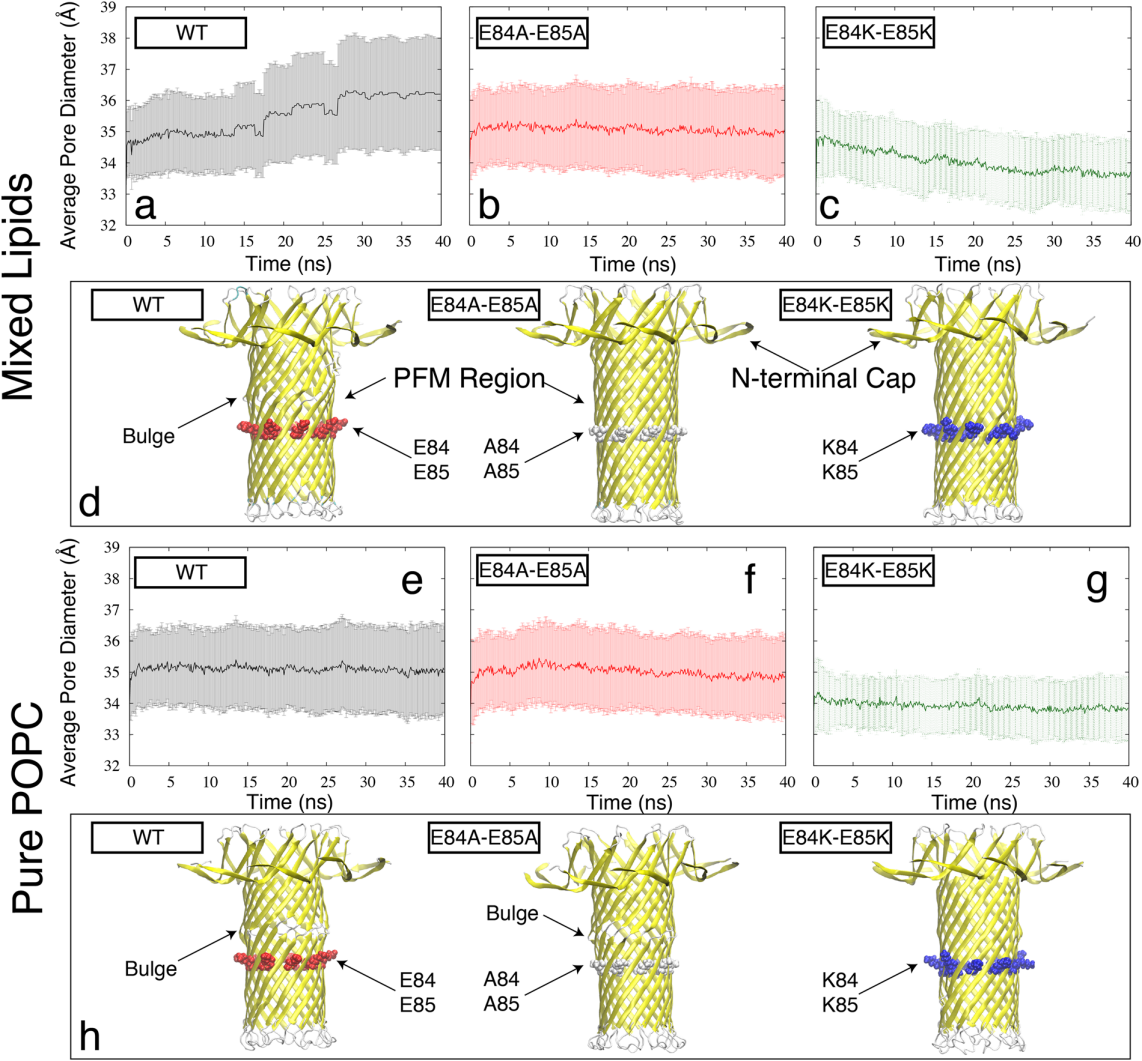
The MD based PFM dynamics quantified by the time series of PFM diameter (see Materials and Methods) for the (**a**) wild-type, (**b**) E84A E85A mutant, and (**c**) E84K E85K mutant lysenin embedded in the mixed lipids and for the (**e**) wild-type, (**f**) E84A E85A mutant, and (**g**) E84K E85K mutant lysenin embedded in the pure POPC lipids. The error bars represent the standard deviation of the pore diameter along the PFM pore. A representative snapshot of the PFM region along with the N-terminal Cap is shown for each system in cartoon representation in panels (**d**) and (**h**), for mixed and pure lipids, respectively.

For each model, the 40-ns MD simulation was analyzed to determine the stability and the diameter of the PFM region. The time series of the average diameter of the PFM β-barrel (see Methods) is reported in Fig. 5a–c (for mixed lipids) and Fig. 5e,f (for pure POPC). In mixed lipids, the trend is different for the three systems including wild-type (reaching an average diameter greater than 3.6 nm), E84A E85A mutant (staying around an average diameter of 3.5 nm), and E84K E85K mutant (reaching an average diameter smaller than 3.4 nm). Visual inspection of the PFM region in our simulations also indicates a difference in the PFM p′-barrel conformation of the wild-type and mutant proteins, where in the wild-type lysenin PFM a bulge region appears as shown in Fig. 5d, which is not present in the cryo-EM structure. The analysis of the same wild- type and mutant lysenin models embedded in a pure POPC lipid bilayer shows somewhat a similar trend, where the bulge region shows up in the wild-type simulations (Fig. 5h) and E84K E85K mutant has a narrower opening (~3.4 nm average diameter) as compared to the other two mutant models. However, there are notable lipid-dependent behaviors such as the presence of the bulge region in E84A E85A mutant only in the pure POPC, or the lower average diameter of wild-type lysenin in the pure POPC lipids (~3.5 nm) as compared to the mixed lipids (>3.6 nm). Interestingly, no bulge was observed in any of the E84K E85K mutant simulations and the PFM lumen was associated with the narrowest opening in both lipid environments.

**Figure 6:**
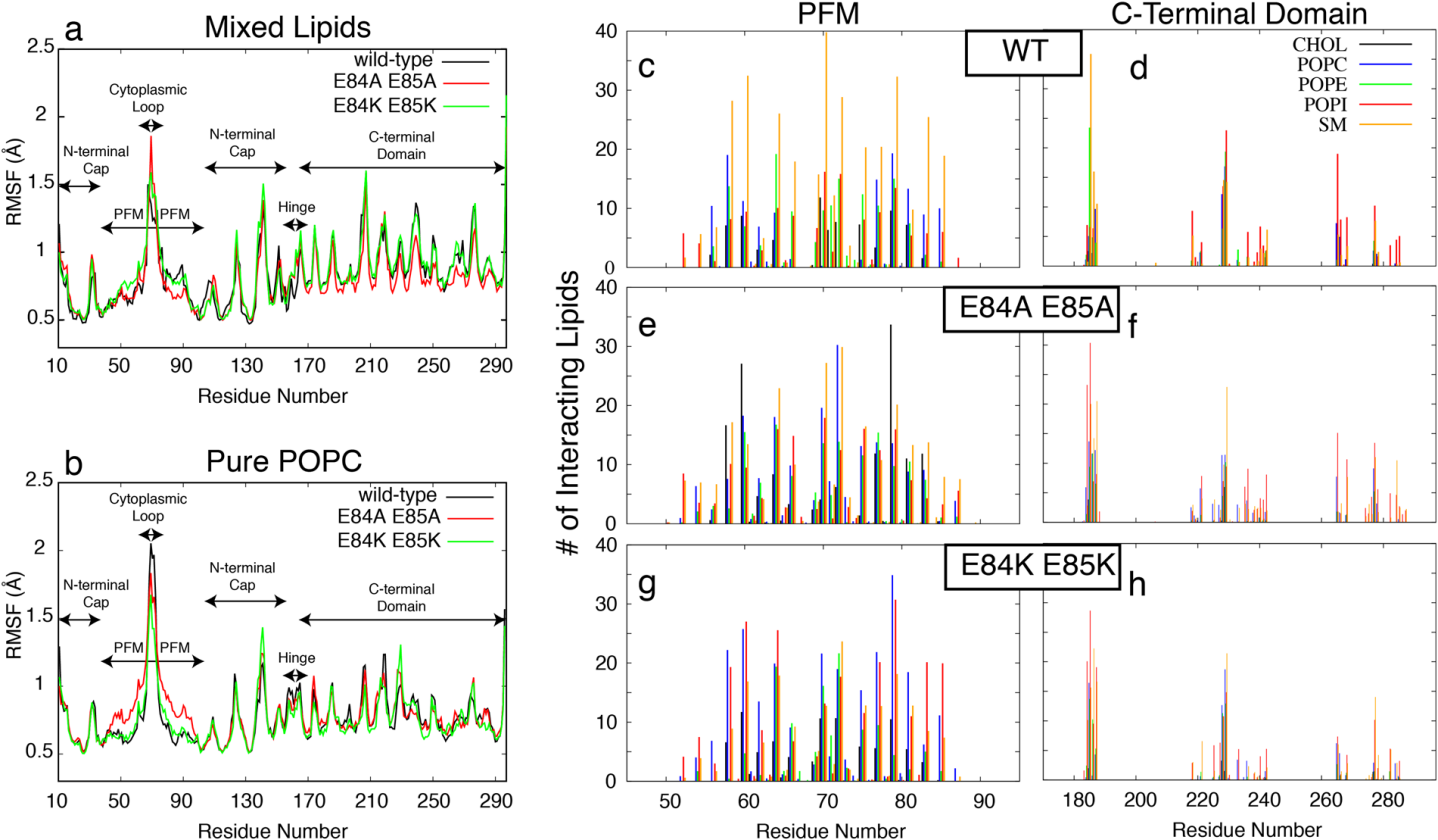
^(**a**,**b**) The RMSF of C^α ^atoms of all residues averaged over 9 protomers calculated from 40-^ ns MD trajectories for the wild-type (black), E84A E85A mutant (red), and E84K E85K mutant (green) lysenin embedded in mixed lipids (**a**) and pure POPC lipids (**b**). (**c**–**h**) The number of lipids interacting with different residues of the PFM region (**c**,**e**,**g**) and the C-terminal domain (**d**,**f**,**h**), averaged over 40-ns MD trajectories are shown for different lipid types including CHOL (black), POPC (blue), POPE (green), POPI (red), and SM (orange).

To investigate the rigidity and flexibility of different regions of protein, we calculated the root-mean-square fluctuation (RMSF) of the C*a* atoms of all residues averaged over 9 protomers (Fig. 6a,b). This represents the average fluctuations of each residue during the 40 ns simulations. All models show similar trends in their fluctuations for different regions. The highest fluctuation is observed for the loops in the cytoplasmic side of the PFM. Another significant fluctuation is observed for the collar region (N-terminal cap domain) with a similar behavior in all models. The PFM region except for its cytoplasmic loop is the most rigid part of the protein. The E84A E85A mutant embedded in the pure POPC lipid bilayer has a distinct fluctuation pattern around the PFM region, which is not observed in any of the other simulations (Fig. 6b).

The C-terminal region is associated with more fluctuations when the protein is embedded in a mixed lipid bilayer (Fig. 6a) as compared to the pure POPC (Fig. 6b). The difference observed for C-terminal domain fluctuations in different lipid environments is potentially due to the interaction of this domain with the lipid bilayer. We have analyzed these interactions as well as the interactions of the PFM regions with lipids and have particularly quantified the distinct interactions of different lipid types with the protein embedded in the mixed lipid bilayer. The average number of different types of lipid interacting with different regions of the protein is shown in Fig. 6. Interestingly, the mutations result in changing the dominant lipids interacting with the PFM region. The dominant lipids interacting with the PFM region of wild-type protein seems to be the SM lipids (Fig. 6c), while cholesterol interactions increase significantly with certain regions of PFM when E84 and E85 are mutated to alanine (Fig. 6d). Charge reversal mutations in the same positions, on the other hand, result in increasing the interactions of negatively charged POPI head groups with not only residues K84 and K85 but also various other residues in the PFM region of E84K E85K mutant.

## Discussion

Using new structural knowledge together with our own electrophysiology data (parts A–C) and computational studies (part D), we propose a model for the mechanism of observed voltage-induced gating of lysenin pores. The model is based on the electrostatic mapping and the structure illustrated in Fig. 1 in which the flexible part of the N-terminal and some zones within the C-terminal domains contain a dominant concentration of positive charges^19^, while the pore-forming module (PFM) side of the protein contains a dominant concentration of negative charges. Accordingly, the entire protein possesses an “effective electric dipole” ($\overrightarrow{{\boldsymbol{P}}}$), in the direction from the PFM to the N-terminal cap domain (collar). This effective dipole plays a key role in the proposed mechanism for gating. In this model, voltage-induced gating of the lysenin channel is due to the electric field alignment of the effective electric dipole resulting in channel closure. More specifically, the mechanism for gating of the pore is due to the electrostatic interaction between the applied electric field ($\overrightarrow{{\boldsymbol{E}}}$) and the effective electric dipole ($\overrightarrow{{\boldsymbol{P}}}$) across the protein^32^, which results in a torque tending to align the effective electric dipole. The dipole aligns potentially because of the movement and flexibility^27^ of the N-terminal cap domain region on the flexible coil of the nonamer. Accordingley, the voltage gating of lysenin originates from this movement which allows an electric field to align the protein effective electric dipole ($\overrightarrow{{\boldsymbol{P}}}$) with the applied electric field ($\overrightarrow{{\boldsymbol{E}}}$). The direction of the electric field determines whether the channel remains in its natural open state (Fig. 1a) or its closed state. The channel stays in its open state for a negative voltage (Fig. 1d) because of the interaction between the external electric field ($\overrightarrow{{\boldsymbol{E}}}$) and the dipole ($\overrightarrow{{\boldsymbol{P}}}$), that acts along the direction of the electric field and in the direction to hold the flexible N- terminal cap (collar) domain in an open position. However, reversing the electric field (shown in Fig. 1d) impacts the electric field-dipole interaction causes the flexible-positively charged N-terminal cap (collar) domain along with the C-terminal domain to move inward in the cis side with possible rotation of the C-terminus. This movement causes a change in its conformation^24^ and blocking of the pathway of the channel resulting in voltage-induced gating of the lysenin pore.

To test the validity of this model, we focused experimentally and computationally on the identification of the flexible region of the N-terminal domain (A to D). Our investigation of our model for voltage-induced gating lead us to prepare the recombinant tagged lysenin. These tags were crucial in preparing the mutant types of the protein (E84&E85). To investigate the role of the negatively charged domain that resides along the channel wall (PFM), we generated a double mutation of the glutamate E84 and E85 first with alanine E84A and E85A and then with lysine E84K and E85K.

**Figure 7:**
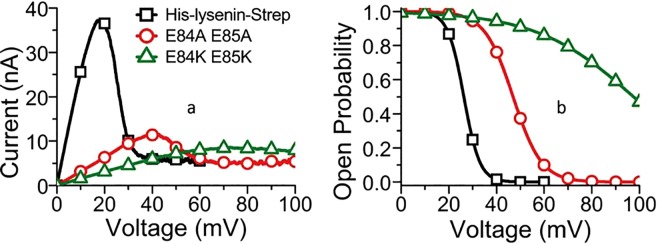
Channels from lysenin generated by recombinant expression respond to voltage stimuli. (**a**) The recombinant, unmodified lysenin yields an I-V curve identical to the wild-type. Mutations with alanine (⚬) caused the voltage gating to shift to higher voltages compared to the expressed lysenin protein with tags (◻). Mutations with lysine (Δ) caused a shift in voltage gating to even higher voltages. (**b**) The mutations caused a shift in the midpoint voltage toward higher voltages.

Before testing our model by exploring protein modification, we first compared the channel formation and functionality of protein channels produced with recombinant His- and Strep-tagged lysenin with the wild-type (Fig. 2a,b) to ensure that the histidine and streptavidin tags did not interfere with the channel’s formation and functionality. The similar behavior of the wild-type and the recombinant protein indicated that the tags used for purification do not modify the channel functionality and can be used for further investigation. Using experimental conditions identical to those for the expressed E84 E85 lysenin and wild-type channels. All the mutant lysenin insert channels (Fig. 2c,d) and exhibited a gating response (Fig. 7a). To confirm that the observations shown in Fig. 2c,d are that of actual channels and not uncontrolled leakage through the membrane, we examined the current versus applied voltage to observe the voltage gating for a large number of pores. A leakage current, i.e. when the pores are closed, (Fig. 7a) is always present and varies slightly with each membrane, apparently due to the number of incomplete nonamers or due to the increase in the membrane fluidity as the voltage increases. These findings confirm their identity as being channels, especially for the E84K E85K mutant where individual channel insertion was difficult to identify (Fig. 2d). We hypothesized that the E84A E85A mutant would weaken the effective electric dipole of the protein, requiring larger voltages to initiate channel gating. In this case, the reduced charge weakens the effective electric dipole and therefore the electric field-dipole interaction. As expected, an increase in applied voltage is observed for gating. To quantify the gating response of wild-type and mutant lysenin, we calculated the open probability from a fit of the I–V curves to a Boltzmann distribution so we could compare the midpoint voltages, V_1/2_, the voltage where the open probability of the channels is 0.5^33^^(^Fig. 7b^).^ The E84A E85A mutant had a ~110% increase in the midpoint voltage over the wild-type protein and the E84K E85K mutant increased the midpoint voltage by ~310%.

We hypothesized that if the N-terminal cap and the C-terminal domain are flexible and function as a movable gate, then the gating behavior should be sensitive to changes in viscosity. The addition of glucose increased the viscosity, but linearly decreased the solution conductivity ^30^ as predicted (Supplementary Fig. S3). While we cannot completely exclude the possibility of additional glucose electrostatic interactions influencing the kinetics of channel gating, we attribute the increase in channel response time to the increased solution viscosity.

We found that the addition of glucose to the electrolyte solution increased the critical voltages in the I-V curves shown in Supplementary Fig. S2. Our previous work with lysenin channels has demonstrated the relationship between voltage-induced gating and the voltage sweep frequency. When the voltage sweep rate exceeds the time for the channels to achieve equilibrium, a lag occurs between the applied voltage and the measured channel current resulting in an apparent shift in the gating toward higher voltages. To demonstrate this effect, we measured the critical voltage from an I-V curve measured at different sweep rates (Supplementary Fig. S4). We found that decreasing the voltage sweep also decreased the critical voltage. Further reducing the rate lower than 0.66 mV/s didn’t change the critical voltage indicating that the voltage sweep was less than the channel equilibrium rate. Remarkably, an approximately 13% increase in the solution viscosity required a 67% reduction in the voltage sweep rate to balance the channel equilibrium rate suggesting that lysenin channels are quite sensitive to the solution viscosity and supporting our finding of a large increase in time constants over the range of viscosities without excluding the decrease in the solution conductivity over that range. The time constants for channel closing (Fig. 3b) were calculated by fitting the decay in ion current. All of the data could be accurately represented by a single exponential decay (the R2 statistics ranged from 0.970–0.999) suggesting a single closing step, at least within the temporal resolution of the instrument. We expect that additional interactions facilitated by the presence of glucose in the solution would be signified as multi step closing kinetics.

In order to shed light on the mechanistic details of the changes in the electrophysiology of lysenin channel due to charge-reversal or neutralization mutations, we performed all-atom MD simulations of membrane-embedded lysenin channels based on the recent cryo-EM structure of full-length lysenin pore^19^. The results of the MD simulations are generally consistent with the effective electric dipole hypothesis. In both the E84A E85A and E84K E85K mutants embedded in a mixed lipid bilayer like that used in our experiments, the channel lumen was observed to be narrower than that of the wild-type protein. The narrowest lumen observed was associated with the E84K E85 K mutant. In the wild-type protein, on the other hand, a bulge was observed above residues 84 and 85, which is not only absent in the mutant models but also does not exist in the cryo-EM or the crystal structure of lysenin^19,20^. The bulge does not appear in the E84K E85K mutant PFM, which is narrower and less distorted than the other two models. The MD simulation results are thus complemented to the results of the voltage gating. The E84K E85K mutant had a ~310% increase in midpoint voltage over the wild-type model, compared to a ~110% increase for the E84A E85A mutant. This is consistent with the order of average PFM diameter in the three models (>3.6, ~3.5, and <3.4 nm for wild-type, E84A E85A, and E84K E85K, respectively).

The simulations of the same protein models embedded in a different lipid environment (pure POPC) show the key role of the environment in the conformational landscape of the lysenin. It is important to note that both cryo-EM and crystal structure of full-length lysenin were determined in a detergent environment. Therefore, the absence of the bulge region in these proteins and apparent rigidity of their conformational could potentially be due the absence of physiologically relevant lipids in those experimental conditions as lipids and their composition play a crucial role in protein conformation, evidenced by results shown in Fig. 5 for lysenin and elsewhere^34^ for other membrane proteins. The results of the RMSF analysis were consistent with the assumption that the N-terminal cap domain is flexible and functions as part of a movable gate for the lysenin channel. If the simulation time is increased, it may be possible to see an even larger fluctuation for the collar region. The 40 nanosecond simulations are very short as compared to the timescales relevant to the function of protein. However, these short MD simulations are in general agreement with results from the experimental data. Just as with the wild-type lysenin, negative applied voltages were not found to gate the recombinant protein channels. These results support the model based on the interaction of the external electric field with the voltage domain sensor of the dipole resulting in gating of the channel.

The hypothesized channel structure in Fig. 1 provides a simple model of the inserted lysenin channel. Water-soluble lysenin monomers binds to sphingomyelin within the lipid bilayer and form a pore whose diameter is likely determined by the repulsive Coulomb force due to high concentration of fixed negative charges in the pore wall facing the lipids. This is expected based on our model for both E84A E85A and E84K E85K, due to the reduction in the fixed negative charge which results in decreased Coulomb repulsion in the pore wall and thus reduction in the size of the channel lumen. Accordingly, the protein possesses an effective electric dipole ($\overrightarrow{{\boldsymbol{P}}}$) directed from the PFM to N-terminal cap domain, and it plays a key role in the proposed mechanism for gating. An applied electric field in the same direction of the dipole produces a torque on the flexible cap domain resulting the pore to gate. This aspect of the model was tested by changing the viscosity of the surrounding to the protein head causing an increase in the gating response time as expected. The model was further tested by decreasing the charge on the N-terminus (specifically in the PFM).

As predicted, the conductance of the pore has been decreased. It is reasonable to assume that the conductance of the pore is dependent on both the charge distribution of the lumen and its geometry. The OmpF pore, for instance, has been shown to have a pH-dependent conductance^33^ that is likely to be due to the charge distribution of the lumen, which is highly pH-dependent due to the presence of various titratable residues within the lumen in OmpF. In the case of the lysenin pore, however, altering the charge of a few residues on the outer surface of the pore does not seem to be able to change the charge distribution of the inner surface of the pore. We have some computational evidence that the geometry of the pore could be altered by mutating E84 and E85. Our simulations are not long enough to reveal the equilibrium geometry of the pore under different conditions. Instead, these simulations simply indicate that the pore geometry is likely to be altered by the suggested mutations.

Our experimental results were complemented by conducting computational investigation of the structural dynamics of the protein in a similar lipid environment. The lipid bilayer proved to be a key factor in determining the conformational landscape of the protein. *A lipid-dependent bulge* region was observed in the supposedly rigid PFM region of protein, which may play a crucial role in the gating mechanism of lysenin. The PFM dynamics is not only lipid dependent, which may explain why it was not observed in other detergent-based structural studies^19,20^, but also behaves differently in the wild-type, and different mutant types of lysenin, following an interesting trend quite consistent with our electrophysiology data.

Our research focused on understanding the mechanisms responsible for the gating behavior of lysenin channels and the ability to alter the channel properties. Certainly, a whole body of work from different laboratories has contributed to our understanding of both the structure and function of the lysenin channel. Our results add important insights into the channel gating mechanism. While we have addressed many questions, many interesting questions remain. What is the motion that closes the pore? Can we use our model to design the channel to be closed initially? Further studies of the lysenin and other channels are underway to answer these and other questions.

## Methods

### Lipids

Asolectin (Aso) from soybean, lysenin, Sphingomyelin (SM) and Cholesterol (Chol) were purchased from Sigma-Aldrich and Avanti Polar Lipids. Other chemicals were purchased commercially and used without further purification. The lipids were dissolved in n-decane, and deionized water was used for aqueous solutions.

### Solutions

130 mM KCl buffered, 20 mM HEPES at pH 7.0 was used as a supporting electrolyte to observe the bilayer formation and lysenin insertion, and to measure the conductance of lysenin channels under the influence of different voltages.

### Antibody

Strep tag II monoclonal antibody (IgG1) was purchased from Novagen, and Anti- GST (mouse) monoclonal antibody (α-GST) was purchased from Rockland.

### Viscosity

The viscosity of electrolyte solution with increasing concentrations of glucose was measured with an AMVn viscometer (Anton Paar) apparatus at room temperature (~22 °C).

### Wild-type and recombinant lysenin production

Wild-type lysenin (_wt_lysenin) (Sigma- Aldrich) was prepared as a 30 μM stock for electrophysiology studies by dissolving 50 μg of lysenin in 50 μl of 10 mM HEPES/KOH (pH 7.0) containing 30 mM KCl and 50% Glycerol). Recombinant lysenin (_rec_lysenin) was produced in *E. coli* with an N-terminal 6-histidine tag and a C-terminal strep tag II. cDNA coding for _rec_lysenin was made by first synthesizing the lysenin coding sequence (Accession Number D85846) with codons that introduced a 6-His affinity tag to the N-terminus. The DNA synthesis product (GeneArt Gene Synthesis, Life Technologies) was then PCR amplified using a reverse primer that introduced a thrombin cleavage site and strep tag II at the C terminus as indicated below. The resulting PCR product was cloned into pQE-80L (Qiagen) using BamHI and HindIII sites to generate _rec_lysenin. Primers for PCR amplification of DNA were purchased from Integrated DNA Technologies. PCR amplifications were performed with Apex DNA polymerase (Genessee Scientific), and PCR products were restricted and ligated with enzymes purchased from New England Biolabs. All reagents, enzymes, and standards were purchased commercially. The construct was sequence verified by Molecular Resource Laboratory,

University of Arkansas for Medical Sciences, Little Rock. The amino acid sequence for _rec_lysenin is shown below. Amino acids corresponding to _wt_lysenin are underlined while those added to the N-terminus including the 6-Histadine tag are bold. Amino acids at the C-terminus corresponding to the thrombin cleavage site (bold italicized) and the strep tag II (italicized) are also indicated.

**MRGSHHHHHH**GSMSAKAAEGYEQIEVDVVAVWKEGYVYENRGSTSVDQKITITKGMKNVNSETRTVTATHSIGSTISTGDAFEIGSVEVSYSHSHEESQVSMTETEVYESKVIEHTITIPPTSKFTRWQLNADVGGADIEYMYLIDEVTPIGGTQSIPQVITSRAKIIVGRQIILGKTEIRIKHAERKEYMTVVSRKSWPAATLGHSKLFKFVLYEDWGGFRIKTLNTMYSGYEYAYSSDQGGIYFDQGTDNPKQRWAINKSLPLRHGDVVTFMNKYFTRSGLCYDDGPATNVYCLDKREDKWILEVVG***LVPRGS****SAWSHPQFEK*-.

BL21 Star (Life Technologies) cells transformed with plasmid coding for _rec_lysenin were grown to mid-log phase and induced with 1 mM IPTG for overnight expression at 25C. The soluble bacterial fraction containing _rec_lysenin was initially purified over TALON Superflow Metal Affinity Resin (Clontech) and further purified using Strep-Tactin Superflow Agarose (EMD Biosciences). The eluted material was then desalted into 2X PBS (280 mM NaCl, 20 mM Na_2_HPO_4_, 36 mM KH_2_PO_4_, 54 mM KCl pH 7.0) before adding an equal volume of glycerol. Protein concentration was estimated by Coomassie Blue staining of purified proteins along with protein standards.

### Lipid membrane, channel formation, and experimental apparatus

The BLM was composed of a lipid solution containing a 10:5:5 weight ratio of asolectin, sphingomyelin, and cholesterol dissolved in 400 µl of n-decane. A planar BLM was formed by painting small amounts of the lipid mixture over a ~70 μm diameter aperture in a thin Teflon (PTFE) film which separated two- 1 ml Teflon (PTFE) reservoirs filled with buffered electrolyte (130 mM KCl, 20 mM HEPES, pH 7.0)^18,35–37^. Electrical connections were established via two Ag/AgCl electrodes embedded in the electrolyte solution on each side of the BLM and connected to the headstage of an Axopatch 200B amplifier (Molecular Devices). Electrophysiology data was digitized and recorded through a DigiData 1440 A Digitizer (Molecular Devices), and further analyzed by using PClamp 10.2 (Molecular Devices) and Origin 8.5 (OriginLab) software packages.

The BLM formation was monitored by measuring its capacitance and leakage resistance, and after a stable BLM was achieved, small amounts of lysenin (~0.3 nM final concentration) were added to the grounded side of the BLM under continuous stirring with a low-noise magnetic stirrer (Dual Dipole Stirplate, Warner Instruments). Lysenin channels were formed by inserting into a planar lipid membrane (BLM) composed of asolectin, sphingomyelin and cholesterol (10:5:5 weight ratio). Protein channel formation was monitored by measuring the ionic currents through the BLM in voltage clamp conditions (−60 mV bias potential, 1 kHz low pass hardware filter).

Stepwise insertion of pores was observed with a steady-state open current signifying the completion of channel insertion^21^. The voltage gating was initiated in the usual manner^38^ by applying a linear voltage ramp of 0.17 mV/s across the membrane until non-linearity occurred. Electro-physiological measurements were conducted to examine ion transport activity of wild-type and mutant recombinant strep-tagged lysenin in which charged residues forming a hypothesized intra-molecular dipole were changed by substitution of alanine for one of the charged groups of the dipole or to lysine for an uncharged group or for a carboxy containing group. Strep tag II Antibody binding to the C-terminus was used to add inertia and viscous drag to effect changes in the rate of voltage gating.

For the viscosity investigations, wild-type lysenin channels were inserted in planar BLMs at room temperature (~22 °C). The viscosity of the electrolyte solution was increased by the addition of small amounts of a 2 M glucose solution in 130 mM KCl, 20 mM HEPES pH 7.0 to both sides of the membrane to avoid differential osmotic effects. After each addition of glucose, we allow the system to equilibrate, signaled by a constant ionic current. The, trans-side of the channels was then biased with a constant potential of +60 mV and the cis chamber was held at ground (1 kHz low pass hardware filter) while the time evolution of the current was recorded.

### Protein structure and charge computations and calculations

The electrostatic representation was prepared from the structure of the lysenin-sphingomyelin complex available from the Protein Data Bank, identifier 5GAQ. The protein structure file was converted into a PQR format to incorporate radii and charge for the electrostatic calculation using the software PDB2PQR^39^. The electrostatic calculations were then performed using the Adaptive Poisson-Boltzmann Solver (APBS) software^40^. Molecular graphic representations were created using Protein Workshop from Protein Data Bank (PDB). The isoelectric point of each variant of lysenin was calculated with ProMoST^41^ (http://proteomics.mcw.edu/promost).

### Molecular dynamics simulation

MD simulations were performed based on the cryo-EM structure of lysenin (PDB identifier: 5GAQ)^19^ CHARMM-GUI^42,43^ was used to generate three different models of wild-type, E84A E85A mutant and E84K E85K mutant lysenin. MD simulations were performed using NAMD 2.11 simulation package^44^ with the CHARMM36 force field^45,46^. The input files for energy minimization, equilibration steps and production run were generated using CHARMM-GUI’s Membrane Builder plugin^43^. A homogenous POPC lipid bilayer (pure POPC) was used for three of the models, and a heterogeneous lipid bilayer (mixed lipids) was used for the other three models, with approximately 800 lipids in the upper leaflet and 800 in the lower leaflet. The mixed lipids mimic the experimental lipid bilayer that consisted of asolectin, cholesterol and sphingomyelin in a 2:1:1 ratio. Asolectin is a mixture of lecithin (mostly POPC), cephalin (mostly POPE) and POPI in roughly equal proportions. Therefore, the simulated mixed lipid bilayer consisted of POPC (34%), POPE (33%), POPI (33%), cholesterol (25%) and sphingomyelin (25%). The models were then solvated in a rectangular water box of size and 150 mM of NaCl ions were inserted into each system using the Monte-Carlo ion placing method^43^. Initially, we energy-minimized each system for 10,000 steps using conjugate gradient algorithm. Then, we relaxed the systems using restrained MD simulations in a stepwise manner (for a total of ~1 ns) using a procedure that is explained elsewhere^42,43^. Production runs were carried out in an NPT ensemble (with constant number of particles, pressure, and temperature) at 303.15 K using a Langevin integrator with a time step of 2 fs and damping coefficient of 0.5 ps^−1^. The pressure was maintained at 1 *atm* using the Nosé–Hoover Langevin piston method^47,48^. The smoothed cutoff distance for nonbonded interactions was set to 10–12 Å, and long-range electrostatic interactions were computed with the particle mesh Ewald (PME) method^49^. The production run for each model lasted 40 nanoseconds, in which the conformations were collected every 2 ps. Simulations were executed on the Stampede2 (Texas Advanced Computing Center) and Razor (Arkansas High Performance Computing Center) supercomputers.

Analysis of the simulation trajectories was carried out using Visual Molecular Dynamics (VMD)^50^. To find the average PFM diameter for every snapshot first we found the closest C*α* atom to the centerline of the pore at any given position z along the membrane normal and defined the pore diameter at z as twice the distance of this C*α* atom from the pore centerline. The average PFM diameter was then defined as the average of pore diameter along the PFM region. The standard deviation was then estimated for each snapshot based on the individual pore diameters measured for different z values.

## Supplementary information

Supplementary Information
