# Supplementary material for: An Effective Electric Dipole Model for Voltage-induced Gating Mechanism of Lysenin
Source: Sci Rep. 2019 Aug 7;9:11440. doi: 10.1038/s41598-019-47725-0 (PMC6686002; doi:10.1038/s41598-019-47725-0)
Supplement: Supplementary file 1 — Supplementary Information [file 41598_2019_47725_MOESM1_ESM.pdf]

An Effective Electric Dipole Model for Voltage-induced Gating Mechanism of Lysenin

Radwan Al Faouri[1*], Eric Krueger[2], Vivek Govind Kumar[3], Daniel Fologea[2], David Straub[4], Hanan Alismail[5], Qusay Alfaori[6], Alicia Kight[7], Jess Ray[8], Ralph Henry[7], Mahmoud Moradi[3], Gregory Salamo[8]

[1]Division of Sciences and Mathematics, University of the Ozarks, Clarksville, AR, 72830 USA
[2]Department of Physics, Boise State University, Boise, ID, 83725 USA
[3]Department of Chemistry and Biochemistry, University of Arkansas, Fayetteville, AR, 72701 USA
[4]Department of Biochemistry and Molecular Biology, School of Medicine, University of Arkansas for Medical Sciences, Little Rock, AR, 72205 USA
[5]King Abdullah International Medical Research Center, Applied Medical Sciences, King Saud University, Riyadh, SA
[6]Department of Biomedical Engineering, University of Arkansas, Fayetteville, AR, 72701 USA
[7]Department of Biological Sciences, University of Arkansas, Fayetteville, AR, 72701 USA
[8]Department of Physics, University of Arkansas, Fayetteville, AR, 72701 USA

*Correspondence: Radwan Al Faouri, Ph.D. Division of Sciences and Mathematics, University of the Ozarks, 415 N. College Ave, Clarksville, AR, 72830 USA, Email: radwanaf@uark.edu

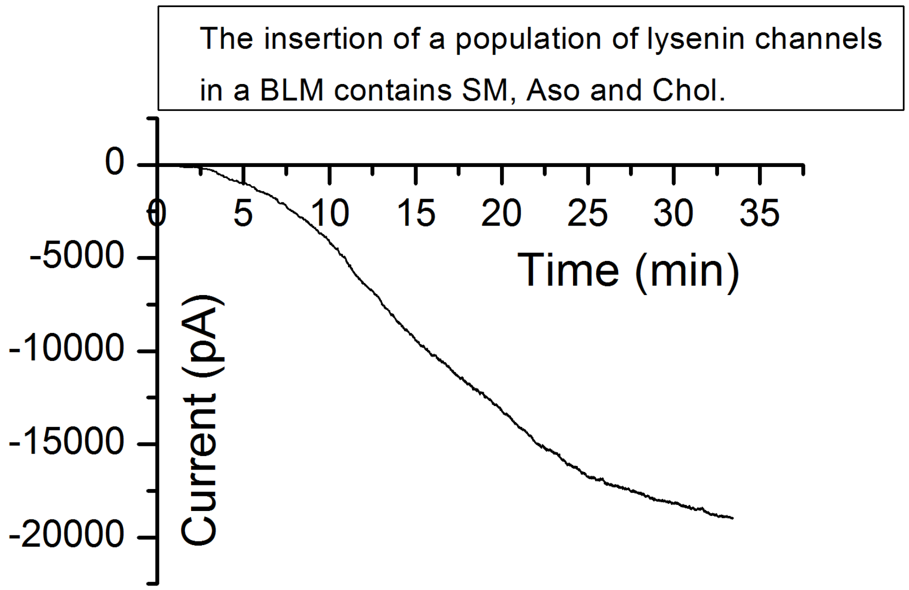

Fig. S1 Current versus time graph shows the insertion of a population of lysenin channels in a BLM

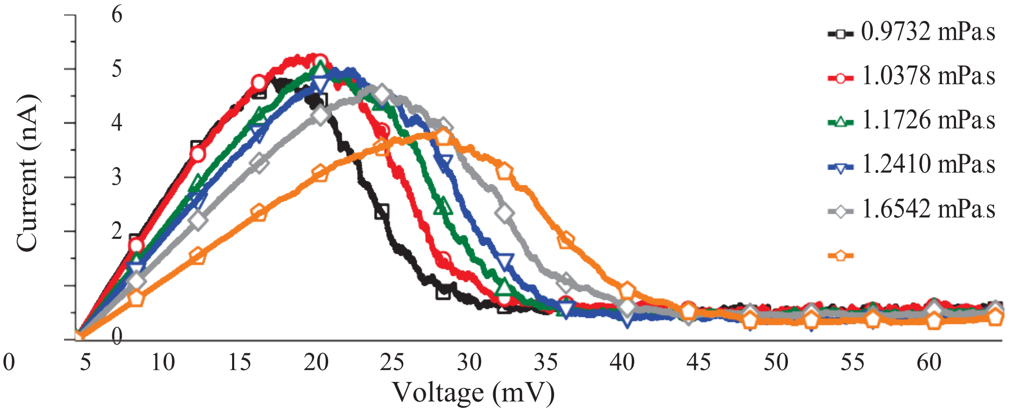

Fig. S2 I-V curves for the lysenin pore in a bilayer lipid membrane as a function of the viscosity of the host ionic fluid.

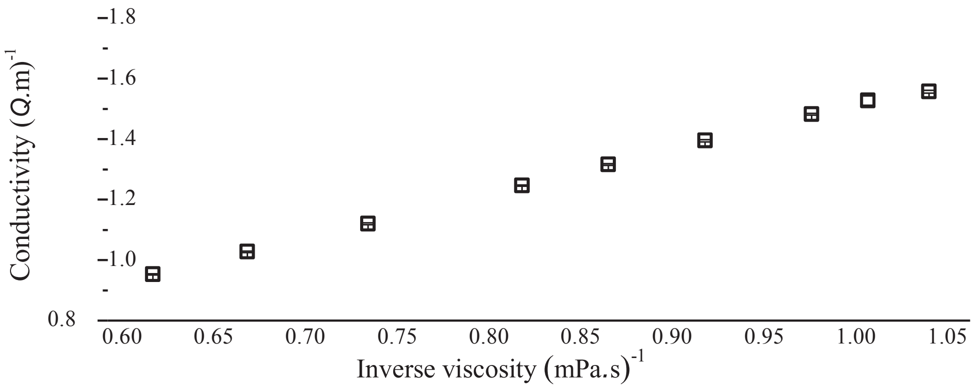

Fig. S3 Conductivity of the host ionic solution verses the measured inverse viscosity of the solution. Note that the conductivity approximately reduces by about two over the viscosity range.

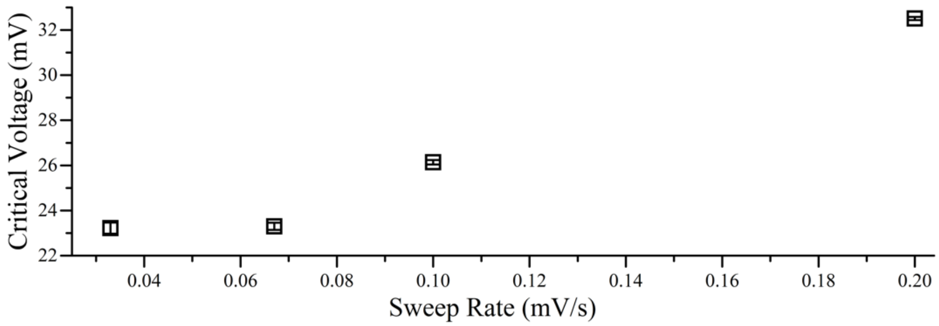

Fig. S4 The critical voltage for decreasing sweep rates for a constant solution viscosity on 1.10 mPa·s.
